# The Life Cycle Assessment of Filamentous Fungi in Pharmaceutical Bioremediation and Wastewater Management: A Critical Review

**DOI:** 10.1021/acsestwater.5c01343

**Published:** 2026-04-21

**Authors:** Brigita Dejus, Emma Luna Tedoldi, Sandis Dejus, Liva Kairisa, Gunaratna Kuttuva Rajarao

**Affiliations:** † Water Systems and Biotechnology Institute, 87254 Riga Technical University, Riga LV1048, Latvia; ‡ Department of Industrial Biotechnology, School of Engineering Science in Chemistry, Biotechnology and Health, 7655 Albanova University Centre, KTH Royal Institute of Technology, Stockholm 114 21, Sweden

**Keywords:** wastewater, fungi, pharmaceuticals, LCA

## Abstract

The effluent from wastewater treatment plants (WWTPs) is a primary source of pharmaceuticals in aquatic systems. The ongoing challenge of managing pharmaceuticals in wastewater highlights the need for advanced treatment techniques and constant monitoring. Despite ongoing research, no current technologies can achieve 100% removal of all pharmaceuticals, while certain compounds persist through both conventional and advanced processes. A possible solution is to enhance WWTPs by incorporating additional tertiary and post-treatment methods such as combining treatment methods, e.g., fungal treatment with activated carbon. Filamentous fungi possess several characteristics including the ability to degrade and adsorb pharmaceutical substances. This article reviews filamentous fungi with conventional and advanced wastewater treatment methods, evaluating their efficacy and sustainability in removing pharmaceutical substances. Additionally, a qualitative environmental impact assessment based on available life-cycle assessment literature on fungal treatment systems for pharmaceutical substances was conducted, highlighting both environmental impacts and potential benefits. To better understand the wastewater management of advanced wastewater treatment methods for pharmaceutical removal, the European legislation governing hazardous substances was analyzed. This critical review aims to provide insights into optimizing fungal-based treatment systems and identify future research directions to enhance the integration of fungal-based methods into existing wastewater treatment plants for pharmaceutical removal.

## Graphical abstract

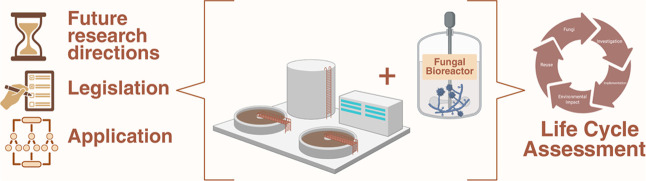

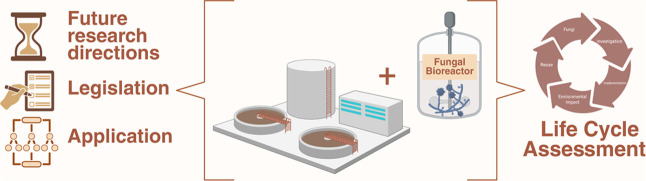

## 1 Introduction

Micropollutants, also referred to as emerging contaminants, are substances that can be bioaccumulative, persistent, and toxic, posing risks to the environment and human health even in trace amounts.^1,2^ This group of emerging contaminants includes personal care products, endocrine disruptors, pesticides, industrial chemicals, and pharmaceutically active compounds (PhACs).^3,4^ Despite being present in concentrations ranging from 1 ng/L to 1 μg/L (in some cases, concentrations can go up to 100 μg/L), research shows that these substances can accumulate in the food chain and impact the environment, fauna, and human health.^5,6^ The relatively low concentrations (i.e., <1 ng/L) and diverse nature of micropollutants complicate their detection and analysis while presenting challenges for water and wastewater treatment processes.^2^ Furthermore, the effluents of municipal wastewater treatment plants (WWTPs) are a significant means of discharging micropollutants into the environment.^7^ The release of treated wastewater containing micropollutants from WWTPs results in detectable concentrations in surface waters, presenting potential risks to aquatic environments.^8^ Thus, a dilution strategy alone is insufficient to mitigate these risks, as substances such as antibiotics and analgesics can still surpass risk thresholds in developing antibiotic resistance in receiving waters.^9^ Conventional WWTPs focus primarily on the removal of organic matter, suspended solids, nutrients, and pathogens from municipal sewage and industrial effluents.^10^ They typically employ physical, biological, and chemical processes, including screening, sedimentation, biological aeration, and disinfection, to achieve wastewater quality suitable for environmental discharge or reuse.^11^ Conventional WWTPs are usually not designed to remove micropollutants^12–14^ because they are generally intended to handle easily and moderately degradable organics, nitrogen, and phosphorus in the milligram per liter (mg/L) range.^15^ However, WWTPs can also serve as a barrier to control the discharge of these micropollutants into the environment.^16^ Thus, monitoring and effectively removing micropollutants from wastewater before discharge remain critical.^17^

The removal efficiency of micropollutants depends on various factors, including WWTP technology, operating conditions, microbial community composition, disinfection methods, chemical structure, and other parameters.^18^ In conventional WWTP systems, the efficiency of removing such compounds varies widely, from negative to complete removal, depending on the influent mass flow of the compounds, the environmental and operating conditions of the treatment scheme, and the compounds’ physicochemical properties (size, concentration, functional group, and polarity).^19^ Many micropollutants have complex chemical structures, which can sometimes include toxic molecules.^20^ For example, in a study by Gaffney et al. (2017), 32 pharmaceuticals were studied, and acetaminophen (55–623 μg/L), metformin (70–325 μg/L), and caffeine (47–273 μg/L) were detected at higher levels in the influent of Beirolas’ WWTP. This study demonstrated that eight pharmaceutical active compounds (PhACs) were not detected (i.e., nimesulide, sulfadiazine, sulfamerazine, sulfamethazine, sulfathiazole, clofibric acid, prednisone, and prednisolone) and four PhACs were completely removed in the WWTP (acetylsalicylic acid, ibuprofen, testosterone, and hydrocortisone), while several PhACs (e.g., acetaminophen, acetylsalicylic acid, metformin, gemfibrozil, bezafibrate, and caffeine) showed significant concentration decrease rates in the WWTP, mainly in the biological treatment.^21^ Similar tendencies were also demonstrated by Bijlsma et al. (2021). The study found that around 50% of the detected 32 PhACs were completely (>80%) or partially (>50%) removed using conventional biological treatment; however, a relatively large number of compounds were not efficiently removed. Most of the concentrations in effluent wastewater did not exceed the average weekly value of 0.1 μg/L, with a few exceptions, including gabapentin (1.1 μg/L), irbesartan (0.13 μg/L), and tramadol (0.37 μg/L). Other compounds, such as clindamycin, levamisole, lorazepam, oxolinic acid, pantoprazole, and venlafaxine, were found at similar concentrations in both influent and effluent wastewater.^22^ Therefore, the capability of treatment methods to remove micropollutants depends on the treatment method, the contact time, and the target pharmaceutical properties (e.g., water solubility, polarizability, hydrophobicity, size, charge, aromaticity, and the presence of specific functional groups).^23^

The possible solution is to enhance WWTPs by incorporating additional tertiary and post-treatment methods.^24^ The efficiency of treatment and thus the extent of pharmaceutical removal by WWTPs can be restricted depending on the compounds’ concentration, chemical structure, solubility, charge, and the existence of viable microorganisms in the WWTP with degradative capabilities.^25^ The latest Urban Wastewater Treatment Directive in the European Union also requires implementation of advanced (i.e., also referred to as quaternary) wastewater treatment processes to limit the loads of pharmaceuticals reaching water bodies.^26,27^ Therefore, there is an urgent need to investigate advanced treatment methods, such as membranes, filtration, ultraviolet irradiation, ozonation, chlorination, advanced oxidation processes, activated carbon, and biological methods (e.g., filamentous fungi), for the effective and cost-efficient removal of micropollutants from wastewater.^28–30^

Advanced treatment methods are used; however, none can eliminate all emerging compounds.^13^ Therefore, new approaches require further investigation. Among them, fungal treatment of wastewater has emerged as a promising technology due to the nonspecific enzymatic systems of lignolytic fungi, which can degrade a wide range of micropollutants even at low concentrations.^31^ Furthermore, this advanced biological method demonstrates sustainability and potential to reduce the use of chemical treatments.^32^ Thus, this review underscores the potential of filamentous fungi as a valuable method for advancing wastewater treatment.

The novelty of this critical review lies in its comprehensive evaluation of filamentous fungi for wastewater treatment with a specific focus on the removal of pharmaceuticals. An environmental impact mapping of a life cycle assessment for fungal treatment systems provides new insights into the environmental impacts and benefits. Additionally, both conventional and advanced treatment methods are assessed for their efficacy and sustainability. To gain a deeper understanding of wastewater management and the relevance of applying advanced treatment methods for pharmaceutical removal, European legislation regarding hazardous substances was examined. Finally, future perspectives have been proposed.

In conducting this critical review, a systematic approach was adopted to ensure comprehensive coverage of the topic. The methodology includes a defined set of keywords related to fungal wastewater treatment from pharmaceuticals, which were used to search Scopus databases^33^ and the AI tool Consensus.App.^34^ The keywords included the following terms: “filamentous fungi,” “white-rot fungi,” “pharmaceutical active compounds,” “advanced wastewater treatment methods,” “latest advancements for wastewater treatment by using filamentous fungi,” “fungal remediation,” “life cycle assessment for fungal wastewater treatment,” and “urban wastewater treatment directive”. After conducting a literature search, the authors selected, reviewed, analyzed, and edited the content to summarize all relevant information regarding the fungi and wastewater treatment. Selection criteria included peer-reviewed articles published within the last ten years to ensure relevance and topicality.

To summarize, by systematically analyzing recent literature and identifying gaps and future research directions, this critical review not only highlights the potential of fungal-based systems but also provides a framework for optimizing and integrating these methods into the existing infrastructure. This approach offers a novel perspective on the use of filamentous fungi in sustainable wastewater management.

## 2 Background of PhACs and Their Bioremediation by Fungi

Pharmaceuticals represent a significant group of emerging pollutants that are essential in both human and veterinary medicine.^35^ According to Patel et al. (2019), micropollutants of PhACs can be broadly categorized into groups such as (i) antibiotics (e.g., amoxicillin, ciprofloxacin, sulfadiazine, sulfamethoxazole, and tetracycline); (ii) analgesics/nonsteroidal anti-inflammatory drugs, including diclofenac, ketoprofen, naproxen, and ibuprofen; (iii) beta-blockers and antihypertensives (e.g., propranolol, diltiazem, and atenolol); (iv) hormonal drugs, such as ethinylestradiol, dienestrol, and mestranol; (v) anticonvulsants, including carbamazepine, felbamate, and primidone; (vi) antidepressants, such as oxazepam, fluoxetine, and diazepam; and (vii) group of antidiabetics (e.g., metformin).^15^ Data from the Organization for Economic Cooperation and Development (OECD) demonstrate that the most frequently consumed pharmaceutical categories include those for the cardiovascular system, the alimentary tract and metabolism, the nervous system, agents targeting the renin–angiotensin system, and blood and blood-forming organs.^36^

The most common pathways of PhACs into the environment are presented in Figure 1. Briefly, the main pathways of PhACs into environments are from pharmaceutical production facilities (industrial WW effluents), and PhAC consumption and extraction to domestic, hospital, and veterinary wastewater.^28,37–39^ Although PhACs are stable, they are not always fully metabolized in both human and animal systems; thus, they can end up in the environment through the wastewater effluent.^26,37,38^ Therefore, the effluent from WWTPs is one of the primary sources of pharmaceuticals in aquatic systems, stressing the inefficiency of conventional treatment in eliminating PhACs.^39,40^

**Figure 1:**
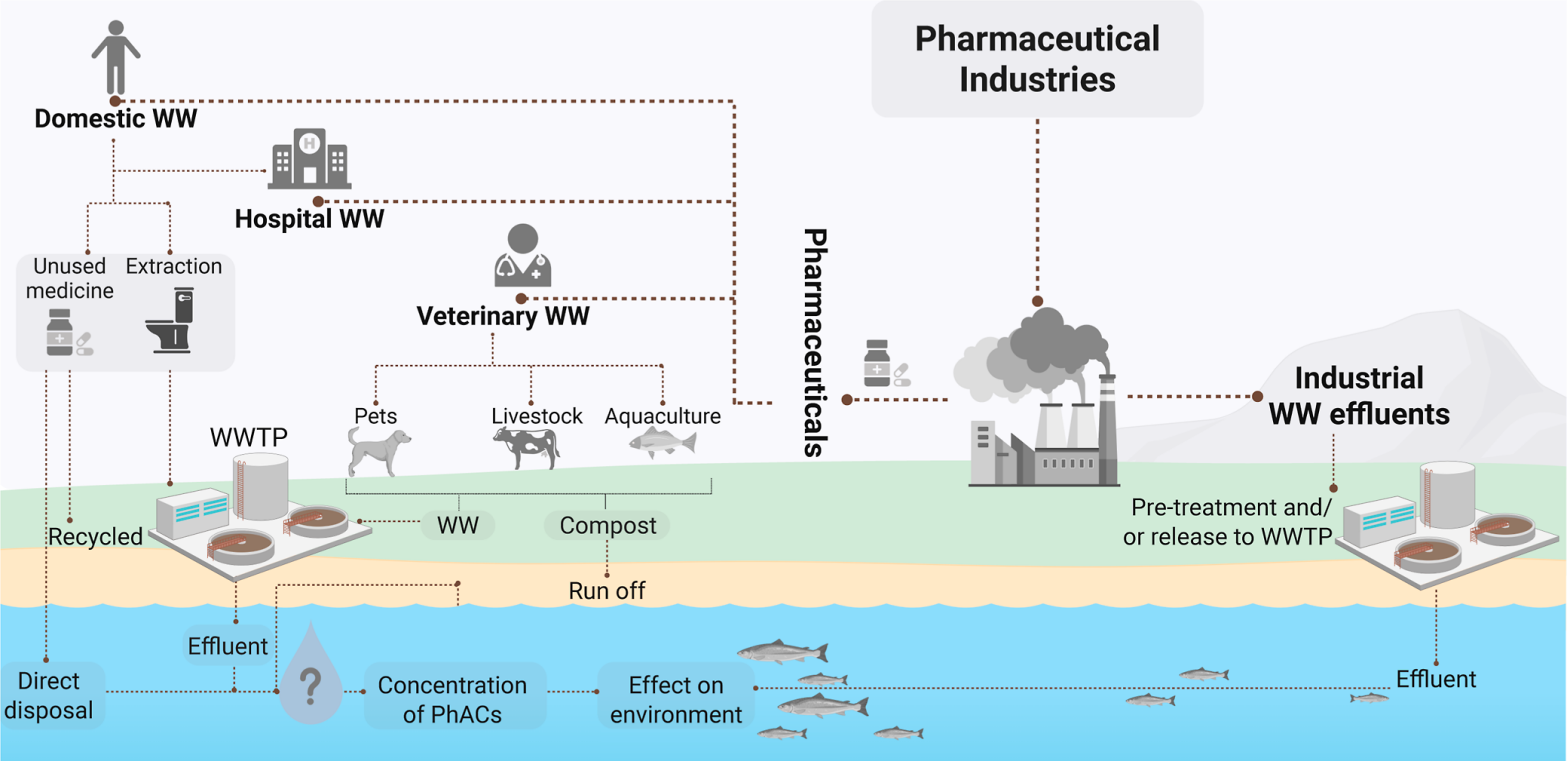
Principal pathways of PhACs into environments.^28,37–39^

Filamentous fungi, particularly white rot fungi (WRF), are essential in wastewater treatment due to their ability to degrade complex organic pollutants. Briefly, the WRF are a diverse group of fungi recognized for their ability to degrade lignin.^41^ The WRF group includes various fungal species primarily belonging to the basidiomycetes.^42^ Notable representatives of this group include filamentous fungi, such as *Pleurotus ostreatus*, *Phanerochaete chrysosporium*, *Trametes versicolor*, *Ganoderma lucidum*, and *Irpex lacteus*.^43^ Over the past decade, these WRFs have demonstrated a significant role in wastewater treatment studies.^44–47^ According to Ferreira et al. (2020), the WRF possesses suitable properties to provide a multidimensional contribution to the removal of PhACs from wastewater.^48^ These include filamentous and macroscopic growth of fungi, the production of enzymes, the capacity for cell wall sorption, the range of value-added products that can be produced, and synergistic outputs when cocultured with other groups of microorganisms (Figure 2). The diverse benefits of fungi include the production of protein-rich single-cell proteins, biolipids, enzymes, and organic acids, which not only enhance environmental sustainability but also foster economic growth.^49^

**Figure 2:**
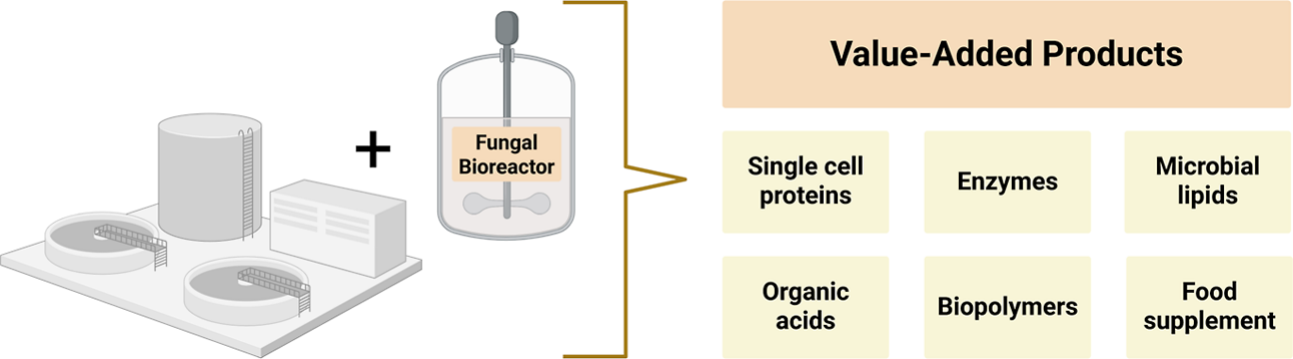
Value-added products from fungal use after wastewater treatment.^48,49^

These characteristics provide an overall advantage over unicellular microorganisms, such as bacteria, yeasts, and algae, due to easier biomass recovery from the medium, lower substrate specificity, higher substrate bioavailability, and potential for a wider range of processes. Fungi also offer an advantage over physicochemical processes for wastewater treatment, as they can produce value-added products with comparatively lower energy and chemical consumption.^48^

The removal mechanisms involved in PhAC treatment with WRF whole-culture can be divided into three main steps, including absorption/adsorption, biodegradation by extracellular enzymes, and degradation by intracellular or mycelium-bound enzymes.^50,51^ Certain fungal strains can also produce biosurfactants (i.e., substances that reduce the surface tension of liquids), which enhance the breakdown of poorly soluble and high-molecular-weight compounds, such as petroleum-derived hydrocarbons, thereby increasing the efficiency of their degradation in wastewater.^52^ The summary of the main mechanisms used by WRF to degrade PhACs is presented in Figure 3.

**Figure 3:**
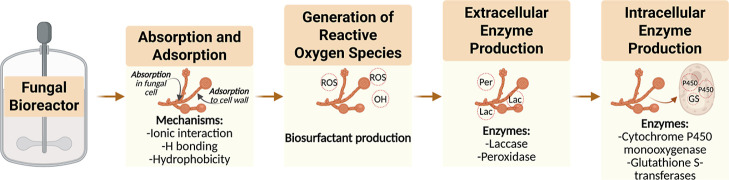
Main mechanism used by WRF to degrade PhACs.^46,52–57^

The primary nonenzymatic mechanisms, adsorption and biosurfactant production, are among the best characterized.^52,58^ The removal mechanisms involve hyphal forms of fungus that can aid in the biosorption process of water purification by allowing PhACs and other chemicals to cling to their surface or be ingested inside the cell, where they are stored rather than transferred in the water.^59^ Enzymatic mechanisms are linked to fungal metabolism, occurring either intracellularly or extracellularly.^52^ Briefly, the intracellular enzymes, such as cytochrome P450 monooxygenase and glutathione S-transferase, are effective catalysts capable of degrading a diverse range of reactions, including aromatic hydroxylation, dealkylation, epoxidation, and dehalogenation^54^ while extracellular enzymes, laccases and peroxidases, stand out as the most important fungal enzymes for degrading PhACs.^46^ The main fungal peroxidases include manganese peroxidase, lignin peroxidase, versatile peroxidases, and dye-decolorizing peroxidases, each having specific substrate preferences.^58^ For instance, manganese peroxidase can oxidize aromatic amines and phenolic compounds by producing reactive free radicals and has been utilized in the removal of polycyclic aromatic hydrocarbons, chlorophenols, and antibiotics.^53^ Overall, the factors that influence mycoremediation include not only fungal species and their ability to produce enzymes but also pollutant structure, culture medium, pH, temperature, and enhancing methods, such as the presence of mediators that affect the removal performance of a WRF.^50,51,56^

To summarize, mycoremediation offers significant advantages as an environmentally friendly approach for degrading PhACs.^55^ Through metabolic processes, it is believed that WRFs can transform these pollutants into simpler, less toxic compounds, thereby mitigating both bioaccumulation and environmental persistence.^55^ Previous studies have demonstrated the efficient removal of PhACs via WRF, achieving removal rates ranging from 78% to 100% in wastewater.^59–61^ Establishing a better understanding of fungal systems and their mechanisms is necessary to gain acceptance as a wastewater treatment technology. For instance, PhACs that enter fungal cells or are present in the environment can potentially affect fungal growth and metabolism.^62^ These compounds may disrupt cellular processes such as enzyme activity or membrane function, leading to altered growth rates or metabolic pathways. Some PhACs, such as carbamazepine at high concentrations, can completely inhibit fungal growth, while others, like diclofenac, triclosan, and naproxen, show a dose-dependent inhibition of radial growth in fungi like *Amylomyces rouxii*.^63^ A previous study also shows that some fungi, such as *T. versicolor* and *Aspergillus luchuensis*, are more tolerant and effective at degrading certain PhACs, while others are more sensitive.^28,64^ Finally, environmental factors such as pH, temperature, and nutrient availability can modulate both fungal growth and the removal efficiency.^51,65^

WRF has the potential to remove PhACs,^48^ highlighting the need for further research to bridge the gap between laboratory findings and a scalable, field-ready solution. Therefore, conducting a life cycle assessment (LCA) of fungal wastewater treatment is crucial for comprehensively evaluating its environmental impacts and application potential.^66^ A clear research gap exists: no direct LCA studies have been published for fungal systems.^67^ LCA can provide valuable insights into the sustainability, efficiency, and potential trade-offs of fungal-based treatment compared to conventional methods.^68^ This approach ensures that fungal technologies not only effectively remove contaminants but also minimize their negative environmental effects throughout their entire life cycle.^66^

## 3 Life Cycle Assessment of Fungi in PhAC Removal in Wastewater Treatment

Life-cycle assessment (LCA) is a standardized methodology (ISO 14040 and ISO 14044, 2006) that provides a comprehensive analysis of the environmental impacts generated by a process or a product over its entire life cycle. LCA is often described as a multicomponent, multistage, and multi-indicator approach that guarantees an exhaustive impact mapping of a process by calculating impacts arising from all the material and energy flows, on every phase of the life cycle, on the whole environment—including climate, resources, and biota.^68^ With the increasing deployment of bioprocesses in a circular economy, LCA is seen as an essential methodology in early-stage process development^66^ to ensure a holistic view of the potential impacts triggered by a new biosolution and limit impact transfers. Several LCA studies in the field of wastewater treatment have highlighted that alternative “low-carbon” processes, which are considered environmentally friendly in themselves, can shift environmental impacts from one indicator (e.g., global warming potential) to another (e.g., ecotoxicity or eutrophication).^69,70^ Therefore, LCA can show great promise in sustainable process development and should be systematically implemented alongside conventional techno-economic assessments, especially in the field of fungal treatment.^68^ However, there is a clear gap in knowledge regarding the application of LCA to fungal wastewater treatment.^67^ When reviewing advances in this field from 2018 to 2023, Malik et al. reported that only 12% of the studies included LCA considerations, primarily through the assessment of effluent toxicity.^59^ No LCA study on fungal advanced treatment for removing PhACs from wastewater has been published to date.

As for advanced treatments for PhCA removal, LCA has become more common, notably for activated carbon, ozonation, and membrane processes with a comparative approach.^71–74^ Nevertheless, these studies show a lack of a standardized LCA framework, which significantly complicates the comparison of results across studies and those of biological methods. For instance, these studies report different functional units, either based on the effluent to treat (e.g., “treating 1 m^3^ of secondary effluent”), based on the treatment efficiency (e.g., “removing pollutant X by 80% in the effluent”) or on a specific threshold for a defined environmental indicator (e.g., “Reduce indicator X below threshold x”). Additionally, studies rely on different LCA methods (e.g., CML, Traci, or ReCiPe), which result in different scoring systems or environmental indicators. Lastly, published LCAs for advanced PhAC removal methods report different scopes, with some taking into account only the building extension and additional bioreactor or basin construction^73^ while others focus on the treatment itself,^71,72^ excluding any construction or dismantling of treatment infrastructures from the modeling. Based on these identified gaps, Table 1 summarizes a proposal for an adapted LCA framework that could be applied to future fungal advanced wastewater treatment studies.

**Table 1:** Summary of an Adapted LCA Framework for Fungal Advanced Wastewater Treatment

elements of LCA framework	considerations for fungal advanced wastewater treatment LCA	references
functional unit	treatment of 1 m^3^ of tertiary wastewater effluent containing pollutants {*x*, *y*, *z*} to achieve a minimum removal efficiency of {*a* %, *b* %, *c* %} using fungal biotreatment.	^75,76^
method	ReCiPe 2016	^76–79^
scope	cradle-to-gate	^76,80^
boundaries	infrastructure construction; operational phase inputs (energy consumption, chemical reagents, and fungal inoculum production); end-of-life, including fungal biomass valorization or disposal; effluent discharge and transformation products.	^81–84^
inventory hotspots	operational energy consumption; infrastructure material (revamping or not, lifespan); chemical inputs; inoculum production; effluent quality.	^76,80–82,84^
allocation rules	water reuse potential: system expansion should credit avoided impacts from conventional water supply.	^76,82,85^
	fungal biomass valorization: should be accounted for through substitution of conventional fertilizers or energy carriers.	
	nutrient removal: functional allocation based on removal efficiency or economic allocation based on regulatory compliance costs.	
sensitivity analysis	energy consumption and electricity mix; targeted pollutant removal efficiencies; infrastructure lifespans.	^75,80,82,83^

Volume-based functional units (FUs; 1 m^3^) are standard in wastewater LCA for comparability. Additionally, removal efficiency thresholds are commonly included to ensure functional equivalence, and pollutant-specific FUs are increasingly used for micropollutant and pharmaceutical studies.^75,76^ The selection of ReCiPe 2016 as the primary LCIA method is justified by several key considerations. As fungal treatment represents an emerging biotechnology, applying an updated harmonized method ensures alignment with current LCA best practices.^77^ The comprehensive characterization of ecotoxicity impacts in ReCiPe 2016, particularly when complemented by USEtox, is critical for evaluating the benefits of pharmaceutical removal.^81^ The advanced treatment context requires a holistic assessment beyond climate change, including eutrophication, resource depletion, and toxicity, all of which are adequately addressed within ReCiPe’s framework. Finally, the increasing adoption of ReCiPe 2016 in wastewater treatment LCA studies facilitates meaningful comparisons across technologies and enhances reproducibility.^75^

Defining appropriate system boundaries is critical to ensuring comprehensive and comparable LCA results in wastewater treatment studies. Following ISO 14044 guidelines and established practices in the wastewater LCA literature,^76,80^ a cradle-to-gate approach is recommended for fungal biotreatment systems. The system boundaries should encompass infrastructure construction, including bioreactor fabrication and additional ancillary equipment, operational phase inputs such as energy consumption, chemical reagents, and fungal inoculum production, as well as end-of-life considerations, including fungal biomass disposal and effluent discharge.^82^ Regarding pharmaceutical fate, the system boundaries must account for both removal efficiency and transformation products, as incomplete degradation may generate metabolites with different ecotoxicological profiles.^81,85^

Based on comprehensive LCA studies of wastewater treatment technologies, five critical inventory hotspots have been identified. First, operational energy consumption represents the dominant environmental burden in biological treatment systems, encompassing aeration, mixing, pumping, and temperature control requirements.^75,80^ Second, infrastructure materials, including bioreactor construction and equipment manufacturing, can account for 10 to 30% of total environmental impacts, depending on the system lifespan.^82^ Third, chemical inputs for pH adjustment, nutrient supplementation, and potential mediators required for enhanced pharmaceutical degradation can constitute a significant hotspot.^81^ Fourth, fungal inoculum production, including cultivation media, sterilization energy, and biomass preparation, represents a specific inventory component unique to fungal treatment systems.^84^ Fifth, waste stream management, particularly residual pharmaceutical compounds in treated effluent, directly influences ecotoxicity and human toxicity indicators.^86^

Allocation procedures for multifunctional processes, such as potential valorization of fungal biomass or water reuse, should be clearly defined following system-expansion or economic-allocation principles, as appropriate.^76,82^ Following the ISO 14044 hierarchy, system expansion through substitution is the preferred approach for multifunctional processes in wastewater treatment LCA.^76^ Three primary cofunctions can require allocation consideration in fungal systems. First, water reuse potential should credit avoided impacts from conventional freshwater supply by expanding the system.^85^ Second, fungal biomass valorization through composting or energy recovery should account for the substitution of conventional fertilizers or energy carriers.^71,84^ Third, simultaneous nutrient and pharmaceutical removal may require functional allocation based on removal efficiency or economic allocation based on regulatory compliance costs when system expansion is impractical.^75,80^ Sensitivity analysis should assess the impact of allocation choices on final results, with transparent documentation of allocation factors and underlying assumptions essential for reproducibility.^76^ Three key parameters warrant a systematic sensitivity assessment. First, energy consumption and electricity mix should be evaluated with variations of ±20–50% in energy efficiency and different regional grid compositions, as operational energy typically dominates environmental impacts.^75,80^ Second, pharmaceutical removal efficiency targets (e.g., 50%, 75%, 90%) should be explored to assess environmental trade-offs between treatment performance and resource requirements.^81,86^ Third, infrastructure lifetime ranging from 15 to 30 years significantly affects the amortization of construction impacts.^82^ Monte Carlo simulation should be employed to propagate uncertainties and provide confidence intervals for results.^87^

Given that available data on the environmental performance of advanced treatments depend on methodological choices and system boundaries and given the scarcity of information on fungal treatments, a quantitative comparison between fungal and conventional advanced processes cannot be reliably performed. However, by integrating data from several sources, a qualitative comparison can be proposed. Figure 4 is a hypothesis-driven impact mapping suggestion for an industrial-scale fungal advanced treatment.

**Figure 4:**
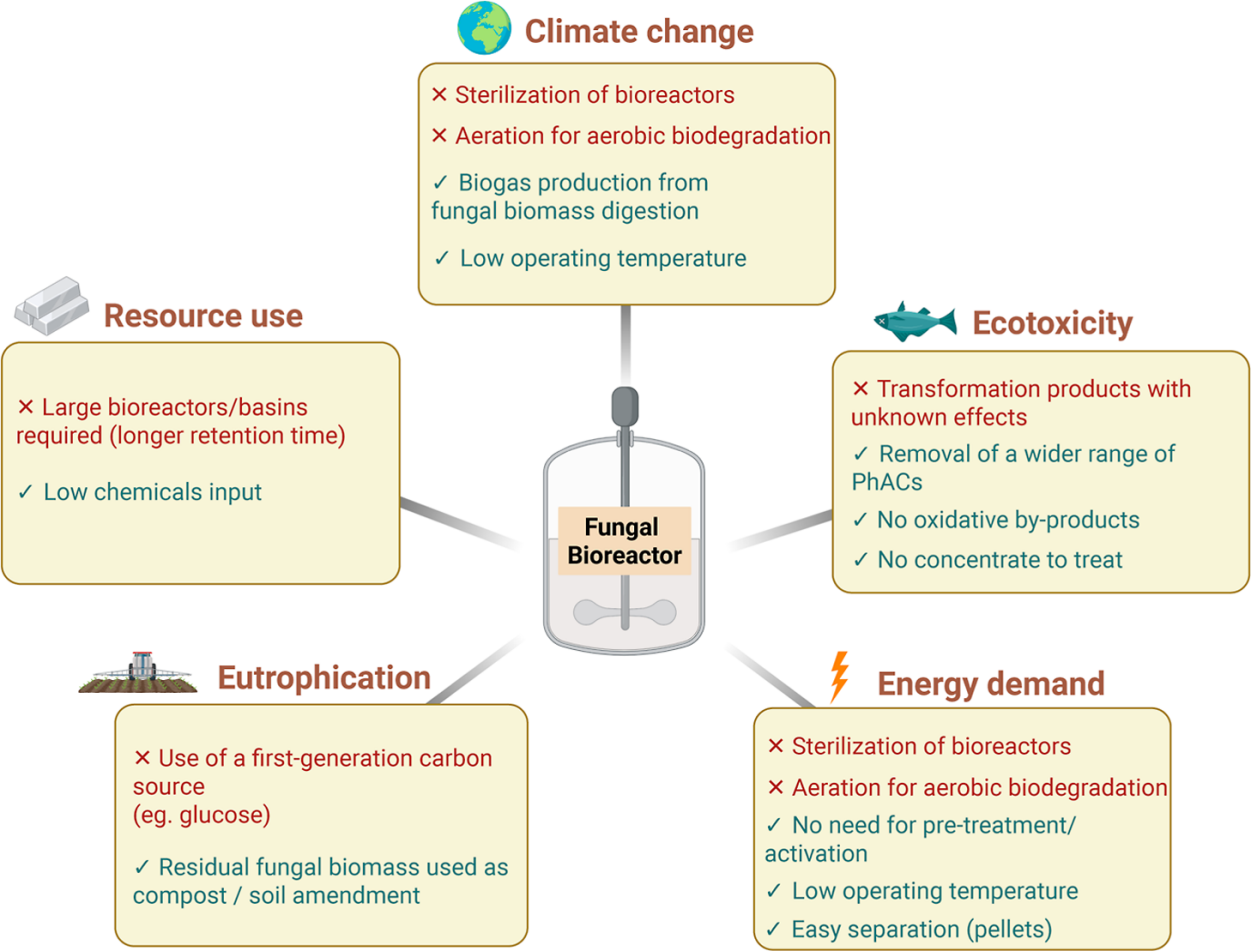
Hypothesis-driven impact mapping of fungal treatment as an advanced treatment for PhAC removal.^72,73,88^ The five impact categories relate to indicators from the product environmental footprint (PEF) methodology of the European Commission, including climate change, freshwater ecotoxicity, human toxicity, use of mineral and fossil resources, and freshwater and terrestrial eutrophication. Energy demand is not a standardized LCA indicator but is still widely used in most LCA studies to quantify the “energy footprint” of products or processes.

Filamentous fungi exhibit high efficiency in removing PhACs, resulting in lower loads in the final effluent discharged into water bodies, which in turn reduces toxicity to freshwater and marine organisms as well as to humans. Several studies have assessed the toxicity of effluents to a wide range of PhACs and personal care products using the UseTOX tool (UNEP’s Life Cycle Initiative),^72,73^ revealing a correlation between the ecotoxicological impacts of effluents and their removal efficiency. However, this statement should be balanced, as incomplete fungal mineralization could yield transformation products and new chemical structures with potential adverse effects on organisms.^88^ Therefore, further investigation is needed to elucidate the ecotoxicity of a fungal-treated effluent.

One additional advantage of a fungal-based approach is the mild operating conditions, which are generally correlated with lower energy consumption compared to oxidative processes or activated carbon, as carbon activation is usually performed at high temperatures and pressures.^41,49^ In addition, fungal pellets have been shown to exhibit high settleability and are easily separated from the effluent;^89,134^ therefore, low-energy separation processes could be efficient at large scales. Nevertheless, aeration and sterilization of bioreactors were identified as energy-intensive processes, with the latter considerably hindering the scale-up of pure-culture fungal systems.^41,57^

Regarding the resource efficiency, due to the relatively slow kinetics and therefore high retention times required for fungal-based treatments, large basins would be necessary,^90,91^ which would increase the resource footprint of the process compared to other advanced treatments when considering the building or extension of the treatment facility. In addition, despite the mild operating conditions reducing the use of harsh chemicals, the carbon source consumption required to maintain fungal growth and activity can have a significant impact on eutrophication, as the upstream burdens associated with agriculture and the use of fertilizers to produce starch-derived glucose are typically considered in LCA studies.^67^

Lastly, residual fungal biomass can be converted into valuable bioproducts or green energy in an integrated biorefinery approach. Therefore, the end-of-life stage of a fungal treatment would considerably reduce the environmental scores of the whole process, especially global warming potential and fossil resources (if residual biomass is converted to biogas or biodiesel, substituting fossil-based energy) or eutrophication (if residual biomass is converted to compost, substituting conventional fertilizers).^67,92,93^ Only one study reports a comparative LCA on fungal treatment and another advanced process for wastewater treatment.^67^ Although this study focuses on the removal of Gray Lanaset G textile dye, rather than PhACs, it provides valuable insights into the comparative sustainability performance of fungi *T. versicolor* and granular activated carbon as advanced treatments for an industrial effluent (Figure 5; the scores were normalized with ReCiPe 2010 normalization factors for improved clarity of the figure).

**Figure 5:**
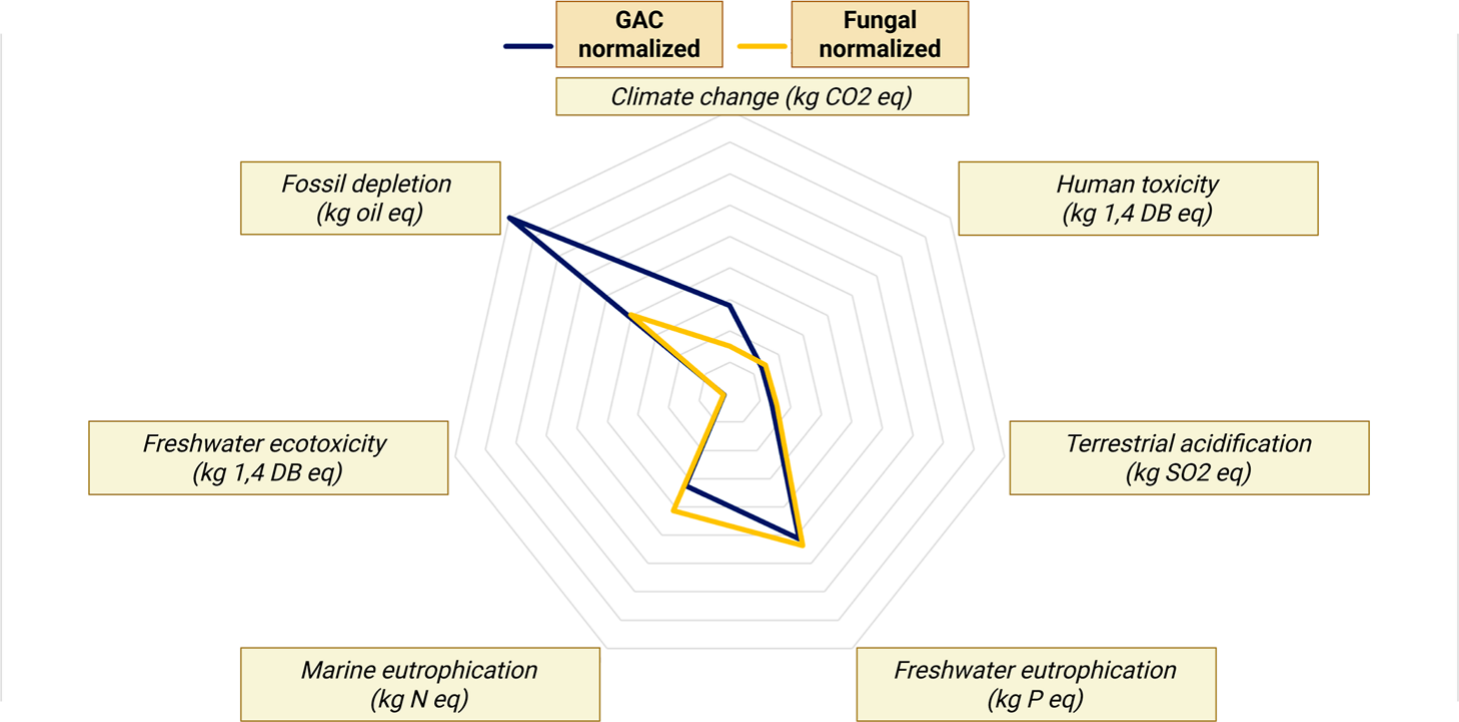
Compared environmental impacts for Gray Lanaset G textile dye removal.^67^

The study highlights the consequent electricity consumption for sterilization and aeration of the bioreactors, reaching between 37% and 97% of total LCA impacts depending on the studied scenarios. Generally, the fungal process performs better in terms of climate change and fossil depletion, notably because the production and regeneration of GAC are very energy intensive. However, the LCA analysis shows that the impact on water eutrophication is higher for the fungal treatment, due to the first-generation glucose modeled in the LCA and its subsequent agricultural burdens (Figure 5). Based on these results, ecodesign strategies, such as in situ cogeneration of steam for sterilization or the use of a second-generation carbon source, can be suggested. Switching to nonsterile environments with fungal consortia was also highlighted in several studies,^41,57^ combined with smart aeration strategies to reduce electricity consumption in bioreactors.^94,95^

## 4 Comparison of Fungal Bioremediation versus Advanced and Conventional Wastewater Treatment Methods

Advanced wastewater treatment technologies are crucial for addressing the limitations of conventional treatment methods, particularly in removing PhACs and other hazardous substances.^96^ The advanced technologies can offer improved efficiency and effectiveness in treating wastewater, making them essential for environmental protection and resource conservation.^97^ Advanced wastewater treatment technologies, including membrane filtration (e.g., ultrafiltration, reverse osmosis), advanced oxidative treatment (e.g., ozonation, photocatalysis), adsorptive (e.g., activated carbon), and biological (bacteria, algae, fungi), present several advantages over conventional treatment methods (Figure 6).^13^

**Figure 6:**
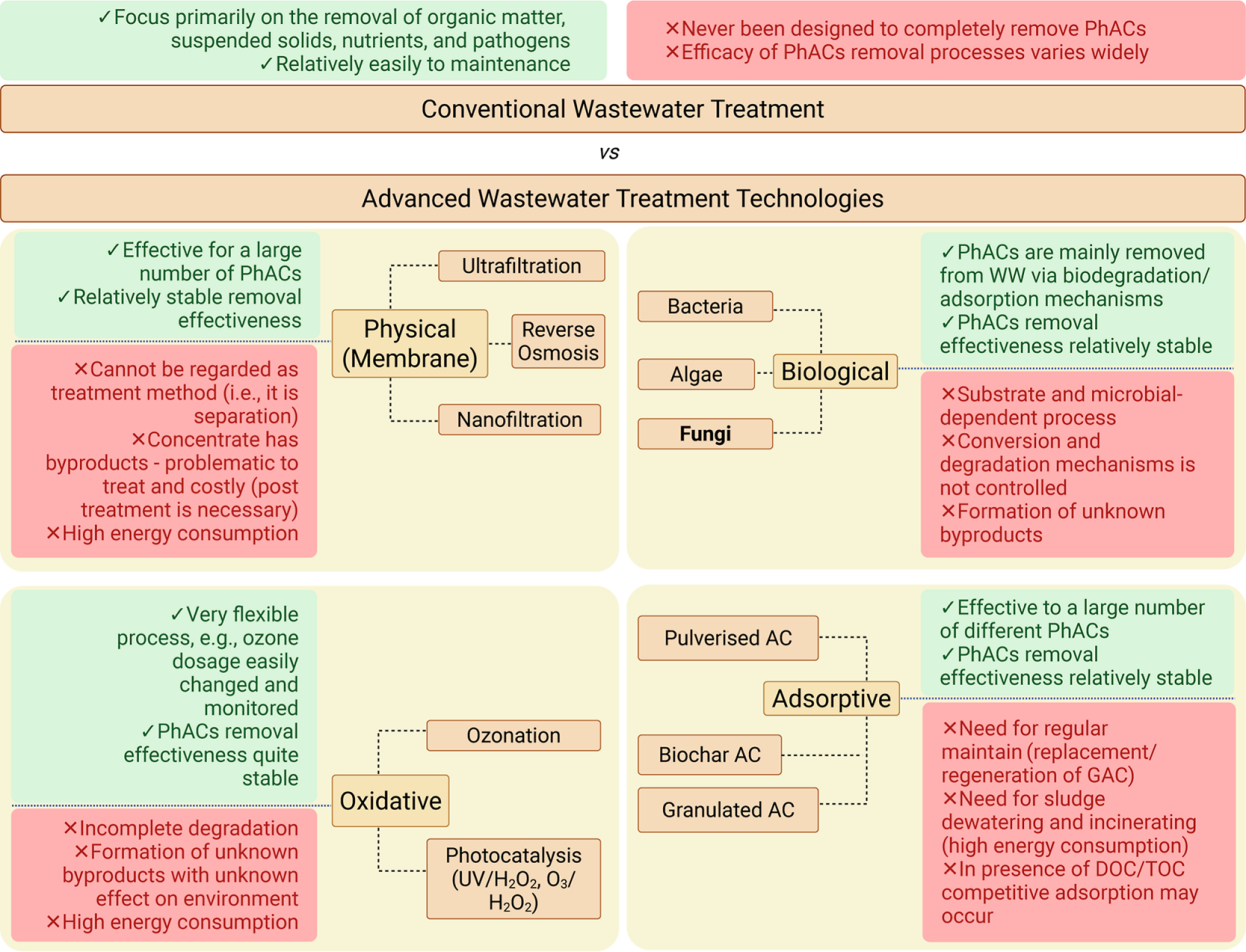
Schematic characterization of classical biological treatment versus advanced treatment for wastewater, including advantages and disadvantages of each technology.^13,14^

Briefly, advanced treatment technologies are characterized by improved treatment performance, allowing for the effective removal of a broader spectrum of pollutants and hazardous substances.^98,99^ The effectiveness of advanced oxidation processes (AOPs) is mainly due to the formation of reactive oxygen species, such as hydroxyl radicals (HO^•^), which subsequently oxidize the organic content of water samples.^99^ AOPs include solar, UV-A, and UV-C photolysis and photocatalysis, usually accelerated by adding titania (TiO_2_) (i.e., heterogeneous catalysis), hydrogen peroxide (H_2_O_2_), and/or iron (Fe^2+^) to form the photo-Fenton reagent (i.e., homogeneous catalysis).^100^ AOPs are energy intensive with high operating costs and an elevated environmental footprint. In contrast, solar photo-Fenton AOPs have high chemical demand and generate residual fluxes with negative environmental impacts, such as sludge contaminated by metal ions and exhausted solid catalysts.^101^ For AOP technology to reach prototype-scale applications, Foteinis et al. (2018) have stated that AOP technology must be environmentally acceptable. Therefore, to overcome these challenges, recent advancements in AOP technology have focused on improving cost efficiency, environmental sustainability, and scalability.^102^ One promising approach is the utilization of renewable energy sources, particularly solar energy, to drive AOPs.^103–105^ For instance, Maniokova et al. (2020) have studied homogeneous solar-driven AOPs, namely, H_2_O_2_/sunlight, solar photo-Fenton (Fe^2+^/H_2_O_2_/sunlight), and solar photo-Fenton with ethylenediamine-N,N′-disuccinic acid (EDDS) complex (Fe^2+^/H_2_O_2_/EDDS/sunlight), and compared them to a new heterogeneous process (supported nitrogen-doped TiO_2_ (N–TiO_2_)/sunlight), with the aim of contributing to fill the gap between lab-scale tests and full-scale applications as well as to provide a sustainable solution for tertiary treatment in small urban wastewater treatment plants. Process efficiency was evaluated in terms of the degradation of a mixture of three pharmaceuticals at an initial concentration of 200 μg/L. Solar-driven photo-Fenton with the chelating agent EDDS was found to be the most effective process.^106^

Regarding cost evaluation, Mouset et al. (2021) have proposed a systematic investigation by introducing a novel criterion, namely, the accumulated oxygen-equivalent chemical-oxidation dose (AOCD), to systematically compare the diverse AOPs available: ozonation, H_2_O_2_ photolysis, Fenton, photo-Fenton, electroFenton, and photoelectro-Fenton (paired with anodic oxidation, for the latter two). Results demonstrated that among the AOPs, electro-Fenton was the most cost-effective (108–125 Euros per m^3^), notwithstanding the mineralization target (50%, 75%, and 99%), owing to its electrocatalytic behavior.^107^ However, Mahbub and Duke (2025) have emphasized that the laboratory-based oxygen-equivalent chemical-oxidation dose (AOCD)/oxygen-equivalent chemical-oxidation capacity (OCC) cost of different AOPs is unable to predict the industry-scale cost of corresponding AOPs correctly. Additionally, the social, environmental, economic, and greenhouse gas emission impact of large-scale AOP plants needs to be assessed as part of their feasibility studies, which cannot be estimated from laboratory-scale cost investigations.^102^

Activated carbon (AC) is a porous, carbon-rich material widely used in wastewater treatment plants to remove hazardous substances due to its simplicity, flexibility, and reliability.^108^ Key operational parameters include breakthrough capacity and operating time, with theoretical lifespans varying depending on whether the targeted compounds are hydrophobic or hydrophilic.^109^ For instance, Nam et al. (2014) demonstrated that the adsorption of micropollutants, especially hydrophilic compounds, was affected by pH. Further research is required to investigate the AC physicochemical properties, viability, and regeneration capacity, with the aim of enhancing the compatibility and mechanical stability of the compounds for wastewater treatment.^110^ Additionally, regular replacement or regeneration of the AC is necessary to maintain its effectiveness, and additional processes, such as sludge dewatering and incineration, which consume significant energy.^103^

Membrane technology has emerged as a favorite choice for reclaiming water from different wastewater streams for reuse; however, significant challenges, particularly membrane fouling, hinder its efficiency and increase operational costs.^111^ For instance, operating these membrane systems requires skilled professionals who can competently manage several critical tasks: accurately documenting system data such as membrane cleaning and replacement schedules, monitoring operational indicators like flow rate, pH, pressure differences, turbidity, conductivity, and routinely assessing the quality of feed, permeate, and concentrate streams.^112^ Proficient analysis of this monitored data is essential for the timely initiation of repairs or corrective maintenance and for predicting the types of fouling, as well as determining the optimal schedule for cleaning or replacing membranes to maintain the desired permeate flux.^113^ It is essential to note that membrane processes are considered separation methods rather than true treatment solutions, as they do not destroy contaminants but rather concentrate them in a waste stream.^114^ For instance, Soni et al. (2009) define a membrane separation process as one that transforms a mixture into two or more distinct end-use products, which aligns with the function of membrane-based processes. Thus, managing the concentrate generated by the membrane separation process is often problematic due to the presence of byproducts, which are difficult and costly to treat or dispose.^115^ Furthermore, membrane processes are characterized by relatively high energy consumption, adding to operational expenses and environmental concerns.^116^ These factors collectively underscore the complexities and challenges associated with the widespread application of membrane technologies in water and wastewater treatment.^117^

To summarize, AOP, AC, and membrane processes can operate efficiently under variable wastewater quality conditions and feature shorter operational cycles, making them adaptable to diverse treatment demands.^13^ Additionally, AOP and membrane technologies tend to produce fewer harmful byproducts, further enhancing their suitability for a wide range of environmental and industrial applications.^103,116^ However, despite their promising capabilities, AOPs are often challenged by high material costs and significant energy requirements.^118^ These factors create barriers to widespread adoption and raise concerns about the economic feasibility of implementing these technologies on a large scale; furthermore, the efficacy of AOPs is frequently influenced by the pH of the water being treated, with many processes functioning optimally only at acidic or neutral pH levels.^99^ According to Hubner et al. (2024), to address these challenges and further advance the field, future directions need to focus on optimizing catalysts and operational parameters to make AOPs more cost-effective and suitable for industrial applications. There is also significant interest in the development of hybrid systems, such as coupling photocatalysis with a membrane system, which may offer enhanced treatment efficiency and enable the removal of an even wider array of pollutants.^119^

Additionally, ongoing innovations in membrane technology, including advancements in materials and system design, are expected to contribute to greater process efficiency while also reducing operational costs.^111^ Many studies report excellent performance of their treatment methods, highlighting the versatility of these processes; however, there is a lack of suitable approaches to compare the removal efficiency and operating costs of different methods under the same conditions.^107^ Therefore, only a few studies provide a comprehensive cost evaluation of advanced methods. For instance, one of these studies is by Utku et al. (2021), which examined the five-year operating costs of nanofiltration (NF) and granular active carbon (GAC) adsorption processes. The study showed that the investment cost of the GAC adsorption process is approximately $1.8 million more than that of the NF membrane process. The operation and maintenance costs of the NF membrane process are $1.1 million more than those of the GAC adsorption process. The operation and maintenance cost of GAC adsorption and NF membrane process is almost 5 times expensive than the conventional treatment process.^120^

In this context, the search for environmentally friendly and low-cost technologies is of great importance. For example, bioremediation can be a viable alternative to AO, AC, and membrane treatment technologies.^121^ The most common organism used to remove organic contaminants in biological wastewater treatment systems is bacteria.^122^ White-rot fungi (WRF), on the other hand, have been successfully demonstrated to have the ability to remove various organic pollutants, including PhACs.^123^

## 5 Legislation Aspects for Advanced Treatment Methods and PhACs

In wastewater management, legislation serves as a fundamental pillar in protecting the aquatic ecosystems and setting baselines for sustainable development.^124^ Regulatory frameworks establish standards that mitigate pollution, protect natural resources, and ensure public health.^125^ These legal instruments not only provide guidelines and enforceable measures but also drive innovation and accountability across industries, fostering a collective responsibility toward environmental stewardship.^126^ For instance, the Urban Wastewater Treatment Directive (UWWTD) is a crucial European regulation that aims to manage the collection, treatment, and discharge of urban wastewater.^127^ The primary goal of the UWWTD is to protect the environment from the negative effects of urban wastewater by setting standards for its monitoring, treatment, and discharge.^128^ The legislation regulating wastewater collection and treatment is primarily based on the “Polluter Pays” and “Control-at-Source” principles, which establish the responsibilities for minimizing the pollution of wastewater systems and, consequently, the environment^129,130^

Under recent proposals, the UWWTD has begun to incorporate the management of micropollutants, including pharmaceuticals (e.g., carbamazepine, diclofenac, metoprolol).^128,131^ The inclusion follows updates to the Water Framework Directive, which previously listed several pharmaceuticals (e.g., azithromycin, carbamazepine, clarithromycin, erythromycin, and ibuprofen).^132^ The new directive aims to establish regulatory targets for contaminants of emerging concern, which include a variety of pharmaceuticals and personal care products.^133^ The UWWTD also highlights the need for advanced treatment.^131^ This additional stage is required for WWTPs serving urban agglomerations of at least 150,000 person equivalents (p.e.), or 10,000 p.e. in specific areas.^134^ According to UWWTD, these treatment methods must achieve a minimum of 80% reduction in micropollutants, and the percentage of removal shall be calculated based on the dry weather flow.^128^ Theoretical studies show that the full implementation of the revised UWWTD will lead to a 40%–69% reduction in PhACs emissions from WWTPs.^127,135^ Treatment methods such as ozonation, activated carbon, and membrane-based technologies are considered effective for this purpose.^29^ However, the implementation of advanced treatment technologies requires substantial financial investments, e.g., for installation and operation.^136^ According to the European Federation of National Associations of Water Services, the removal of micropollutants in UWWTPs will lead to an estimated additional annual cost of € 8–256 per capita/year, which is beyond the costs provided by the Impact Assessment; additionally, advanced treatment will increase the climate footprint and require an extra energy demand of 20–70%.^137^ Furthermore, there is no clear plan in place to manage these expenses in Europe, raising concerns about how municipalities and industries will cover the associated financial costs. To reduce the potential impact on society via tariffs of public wastewater services, based on “Polluters pay” and “Control-at-Source” principles, UWWTD proposes a new Extended Producer Responsibility (EPR) system for producers of pharmaceuticals and cosmetics to fund the construction of tertiary treatment facilities.^130^ However, numerous ongoing court trials question the efficiency of EPR implementation and its potential impact on the prices of medicines and cosmetics.^138^ Nevertheless, without established funding mechanisms or comprehensive strategies, such as effective EPR schemes, achieving the ambitious targets of UWWTD remains uncertain and complex.^137,139^

Overall, even with the full implementation of the “Control-at-Source” principle and a decrease in the use of pharmaceuticals, it may not reach the environmental protection goals regarding micropollution. Thus, moving toward implementing advanced treatment can be the right direction, as it underscores the commitment to enhancing environmental protection and public health. A wide range of advanced treatment methods has been investigated for removing pharmaceuticals from wastewater.^96,140–142^ However, certain knowledge gaps remain. To fully understand the potential of advanced treatment, it is necessary to address the existing knowledge gaps related to treatment methods, including ozonation, activated carbon, and biological treatment methods (i.e., algal and fungal treatment) by ensuring that the selection of advanced treatment must consider technological, economic, and environmental aspects.^13^

## 6 A Current Status of Fungi Application for PhAC Removal

In the past decade, WRF performance has typically been studied in sterile batches (i.e., flask experiments), as bacterial contamination can significantly impair fungal function. Only a few experiments have been carried out in continuous flow bioreactors under nonsterile conditions.^44,59,89,143^ For instance, Gao et al. (2018) demonstrated in sterile batch experiments that *P. sanguineus* could remove 98.5% ciprofloxacin, 96.4% norfloxacin, 100% sulfamethoxazole, and 100% their mixture through biotransformation within 2 days.^116^ However, the primary drawback of WRF treatment for PhAC removal is the challenge posed by competition from other microorganisms in the bioreactor, which can hinder the successful establishment of the inoculated fungus.^144^ Therefore, Palli et al. (2016) conducted a biodegradation experiment, first in flasks and then in packed-bed bioreactors filled with inert and biodegradable carriers (i.e., straw). Reactor inoculated with *P. ostreatus* attached to a straw worked under nonsterile conditions for three months, showing 30 ± 5% naphthalene sulfonic acid polymers (i.e., polymers that are widely used to produce pharmaceuticals, pesticides, cosmetics, polymers, optical brighteners, dispersants, stabilizers, wetting agents, and construction materials) degradation.^143^ Xueqing et al. (2015) used a bioreactor immobilized with *P. chrysosporium* and operated continuously under nonsterile conditions to treat a synthetic wastewater spiked with naproxen and carbamazepine (1 ng/L each) for 165 days. The results of this study showed that no serious bacterial contamination occurred during the operational period. Naproxen was always removed to an undetectable level, regardless of the experimental conditions, whereas the average removal efficiency for carbamazepine reached approximately 80%. Xueqing et al. (2015) demonstrated that the relatively high removal performance was mainly attributed to the application of the carriers, which provided high efficiency in the transfer of oxygen and nutrients inside the bioreactor. From the fungal immobilization combined with temperature adjustment, the fungal activity, including enzyme production, was protected, and bacterial contamination inside the reactor was effectively suppressed. The enzyme kinetics parameters *K*_m_ and *V*_max_ values for naproxen and carbamazepine were 10.87 mg/L and 2.50 mg/L min and 33.55 mg/L and 3.33 mg/L min, respectively.^145^

Similar tendencies in the removal of PhACs and their interaction with other microorganisms have also been observed by Kresinová et al. (2018), who found that *P. ostreatus* demonstrated efficient biodegradation of endocrine-active metabolites. The data revealed that the fungus can operate under various static and continuous-flow regimes of the bioreactors, and despite decreasing the fungal/bacterial ratio after 7 days of cultivation, the degradation efficiency is not substantially negatively influenced by bacterial microflora present in the wastewater. Moreover, the set of experiments demonstrated the feasibility of scaling up the process. The final tested parameters of the bioreactor at the WWTP locality are still far from practical application in communal wastewater treatment; however, its application can be easily focused on smaller WWTPs or used in the industry or hospitals where PhACs are the main pollutants of WW.^123^ Badia-Fabregat et al. (2017) conducted a study using fungi under nonsterile conditions in a continuous operational mode with two types of real wastewater effluent: a reverse osmosis concentrate from a wastewater treatment plant and a veterinary hospital wastewater. The results showed that the bacteria and fungi that developed inside the bioreactors were quantified and identified, indicating that the failure of some fungal-assisted wastewater treatment processes might be due to the competition exerted by indigenous fungi rather than bacteria, in contrast with the results reported in the literature. Further experiments, i.e., at different nutrient (C/N) ratios and including molecular tools to identify the microbial community, are needed to fully understand the behavior of the inoculated fungus and to optimize the operational conditions.^146^

To summarize, further research is required to investigate the kinetics of fungal cultivation and maintenance in bioreactors.^147^ The added limitation of the fungal strain wastewater treatment process is the washing out of mediators and enzymes with the treated effluent.^51,59^ This presents a particular challenge for enzymatic bioreactors, and research on scaling up enzymatic wastewater treatment remains limited.^148^ Thus, in recent years, several studies have expanded into bioreactor systems, offering insights into process optimization under more practical conditions.^149^ For instance, Mir-Tutusaus et al. (2018) studied three variables for which there was little information in fungal fluidized bed reactor systems removing pharmaceuticals: pellet size, aeration, and C/N ratio. The study revealed that pellet size significantly impacted fungal survival, with a critical diameter of 6.7 mm being proposed; furthermore, 2 mm pellets demonstrated a better removal capacity than 3 mm pellets. A 0.8 min 1 aeration and a 7.5 C/N ratio were chosen for extending the treatment length. Pellet production time was reduced from 5 days to 72 h by increasing the mycelial inoculum amount, which also reduced the risk of contamination during the pellet production phase.^150^ Aside from this study, Espinosa-Ortiz et al. (2016) demonstrated that the airlift reactor is the most promising reactor type for wastewater treatment with fungi due to its ability to maintain pellet shape during long-term reactor operation. Further developments for the full-scale application of these bioreactors depend on maintaining a stable activity of the fungal pellets over prolonged periods, as well as good performance under nonsterile conditions.^89^ However, the transition to real-world applications of WRF remains limited; only a few experiments have been performed at a pilot scale, and there is limited information available regarding full-scale implementation.^144^ The best strategy will depend on the wastewater to be treated, the final use of the treated wastewater, and consequently, the cost of the treatment.^43^

Currently, the application of fungi in real-world wastewater treatment plants is limited, primarily due to the complexities involved in maintaining bioreactors, i.e., the difficulty for the inoculated fungus to successfully compete with other microorganisms growing in the bioreactor and managing the diverse capabilities of fungi in pharmaceutical removal.^146^ Over the past decade, these challenges have resulted in relatively few case studies demonstrating the long-term implementation of fungi for wastewater treatment from PhACs (see Table 2). Almost all studies in Table 2 have demonstrated the use of *T. versicolor* for removing a diverse range of pharmaceuticals. The results also indicate that *T. versicolor* is predominantly employed for treating specific types of wastewater, typically hospital wastewater.

**Table 2:** Case Studies on Fungi and Their Long-Term Implementation in Removing PhACs from Wastewater under Nonsterile Conditions

fungus	wastewater type and reactor type	other parameters	targeted compound	key findings	reference
Trametes versicolor	veterinary hospital wastewater; air-pulsed fluidized bed glass bioreactors.	continuous mode	pharmaceuticals such as ketoprofen, ibuprofen, piroxicam, diclofenac, naproxen, etc.	PhAC removal efficiency achieved in the fungal treatment operated in the nonsterile continuous mode was 44% after adjusting the C/N ratio with respect to the previously calculated one for sterile treatments.	^151^
		nonsterile			
		operation time: 26 days			
		temperature: 25 °C pH: 4.5			
		nutrient supply: glucose and ammonia			
Bjerkandera adusta and Pleurotus ostreatus	industrial wastewater; packed-bed bioreactors.	continuous mode	2-naphthalenesulfonic acid polymers	reactor inoculated with P. ostreatus attached on straw worked under nonsterile conditions for three months showing 30 ± 5% NSAP degradation	^143^
		nonsterile			
		operation time: 100 days			
		temperature: NR^a^ pH: 7			
		nutrient supply: Glucose			
Trametes versicolor	real hospital wastewater, fluidized bed bioreactor and trickling packed-bed reactor.	continuous mode	ibuprofen, ketoprofen, and naproxen.	best results were obtained with the trickling packed bed reactor, which operated for 49 days with high removal values.	^152^
		nonsterile with spiked PhACs			
		operation time: 45 days			
		temperature: 25 °C pH: NR			
		nutrient supply: No			
Trametes versicolor	real hospital wastewater, fluidized bed bioreactor.	continuous mode	analgesics, anti-inflammatories, antibiotics, psychiatric drugs.	successfully treated real nonspiked, nonsterile wastewater in a continuous fungal fluidized bed bioreactor coupled to a coagulation–flocculation pretreatment for 56 days.	^153^
		nonsterile			
		operation time: 29 days			
		temperature: NR pH: NR			
		nutrient supply: glucose and NH_4_Cl			
Pleurotus ostreatus	real communal wastewater, trickle-bed reactor.	continuous mode	endocrine disrupters (EDCs; bisphenol A, estrone, 17b-estradiol, estriol, 17a-ethinylestradiol, triclosan, and 4-*n*-nonylphenol).	fungus was able to operate in the presence of bacterial microflora in wastewater without any substantial negative effects on the degradation abilities; a pilot-scale trickle-bed reactor was installed in a wastewater treatment plant and successfully operated for 10 days, where it was able to remove more than 76% of EDCs present in the wastewater.	^123^
		nonsterile			
		operation time: 10 days			
		temperature: NR pH: NR			
		nutrient supply: NR			
Trametes versicolor with mixed fungal/bacterial community	hospital wastewater, air-pulsed fluidized bed bioreactor.	continuous mode	analgesics, anti-inflammatories, antibiotics, including pharmaceuticals such as carbamazepine and venlafaxine.	this work embodied a 91 day long-term robust continuous fungal operation. The operation was able to maintain an average pharmaceutical load removal of over 70% while keeping the white-rot fungus active and predominant through the operation.	^91^
		nonsterile			
		operation time: 91 days			
		temperature: NR pH: NR			
		nutrient supply: glucose and NH_4_Cl			
T. versicolor	real hospital wastewater; stirred tank, and trickle-bed bioreactors.	continuous mode	16 pharmaceuticals (various classes).	up to 89.8% removal; trickle-bed reactor also reduced toxicity.	^61^
		nonsterile			
		operation time: 13 days			
		temperature: 25 °C pH: not adjusted			
		nutrient supply: NR			
Trametes versicolor with mixed fungal/bacterial community	real hospital wastewater, rotating biological contactors.	continuous mode	antibiotics such as azithromycin, metronidazole, and sulfamethoxazole.	up to 98.4% removal for some antibiotics; stable mixed fungal/bacterial biofilm.	^154^
		nonsterile			
		operation time: 75 days			
		temperature: NR pH: 4.5			
		nutrient supply: NR			

a NR—not reported.

No studies have been published that demonstrate the implementation of fungal bioreactors in real wastewater treatment plant systems for removing PhACs. So far, most studies on fungi for pharmaceutical removal from wastewater typically begin with lab-scale experiments that investigate various fungal strains under sterile or nonsterile conditions to assess their potential in degrading different pharmaceuticals.^155^ However, these findings often cannot be directly applied to real wastewater environments, where nonsterile conditions, harsh environments, and competition with natural microorganisms hinder the successful application of fungi.^146^ By accurately identifying the composition of the wastewater, it becomes possible to select the most effective fungal strain for targeted pollutant removal, optimizing treatment outcomes.^156,157^

Regarding the cost evaluation of the WRF treatment process, many studies have reported that filamentous fungi treatment for wastewater is cost-effective;^41,49,57^ however, there are no comprehensive cost evaluations,^158^ and a significant gap remains in fully understanding the actual costs of this method application in real wastewater systems.^57^ Furthermore, there is currently a lack of standardization in fungal treatment methods, making it difficult to compare results from different studies and hindering the development of best practices and cost evaluation.^49^ This foundational LCA analysis will pave the way for the exploration of the potential of fungal biomass for the removal of pharmaceuticals in real wastewater treatment systems.

## 7 Future Perspectives

The authors propose shifting research strategies to focus more on the requirements and challenges of real-life applications, ensuring that fungal solutions are practical and effective in actual wastewater treatment scenarios (Figure 7).

**Figure 7:**
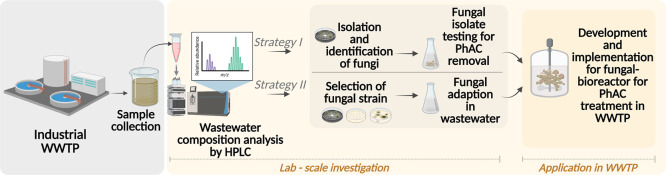
Potential strategies for selecting and adopting fungi for industrial wastewater treatment from PhACs.

Briefly, Strategy I involves analyzing the wastewater composition and isolating fungal strains from the natural environment of the wastewater. Since industrial and pharmaceutical wastewater can be hazardous, microorganisms naturally present are already adapted to these challenging conditions.^159^ For instance, fungi are among the most extreme-tolerant organisms, with highly versatile lifestyles and stunning ecological and morphological plasticity.^160^ Before applying these fungi in bioreactors for treatment, it is crucial to determine their effectiveness in removing specific pharmaceuticals, ensuring their suitability for the task.^161^

Strategy II, like Strategy I, begins by analyzing the wastewater composition to identify the pharmaceuticals that require removal. Based on this analysis, the most suitable fungal strains are selected from the existing literature for their ability to degrade specific pharmaceuticals. To enhance this selection process in the future, developing a comprehensive database that links fungal characteristics with pharmaceutical removal capabilities will be essential, optimizing the selection and reducing the time. Once selected, the fungal strains are introduced into bioreactors for application in wastewater treatment plants. Compared to Strategy II, Strategy I can be more time-consuming and requires greater investment due to the processes of fungal isolation and testing, which can add additional costs. Moreover, not all isolated fungi possess the ability to effectively remove pharmaceuticals. Additionally, when selecting the most suitable fungal strains, it is crucial to test for adaptation time as not all strains can thrive in industrial wastewater environments and compete effectively with natural microorganisms present in the wastewater. To advance the technology to an industrial scale, sterility must be discarded because it is not feasible from both economic and environmental perspectives.^43^

Overall, the ongoing challenge of managing PhACs in wastewater highlights the need for advanced treatment techniques and constant monitoring.^162^ In Figure 8, the authors present a conceptual framework for sampling strategies to monitor PhACs. The aim of the monitoring is to follow the accurate pathways of PhACs in WWTPs and provide data on the removal efficiency of these substances during the treatment process.^163^ Additionally, possible strategies for implementing advanced treatment in WWTPs are also proposed in Figure 8.

**Figure 8:**
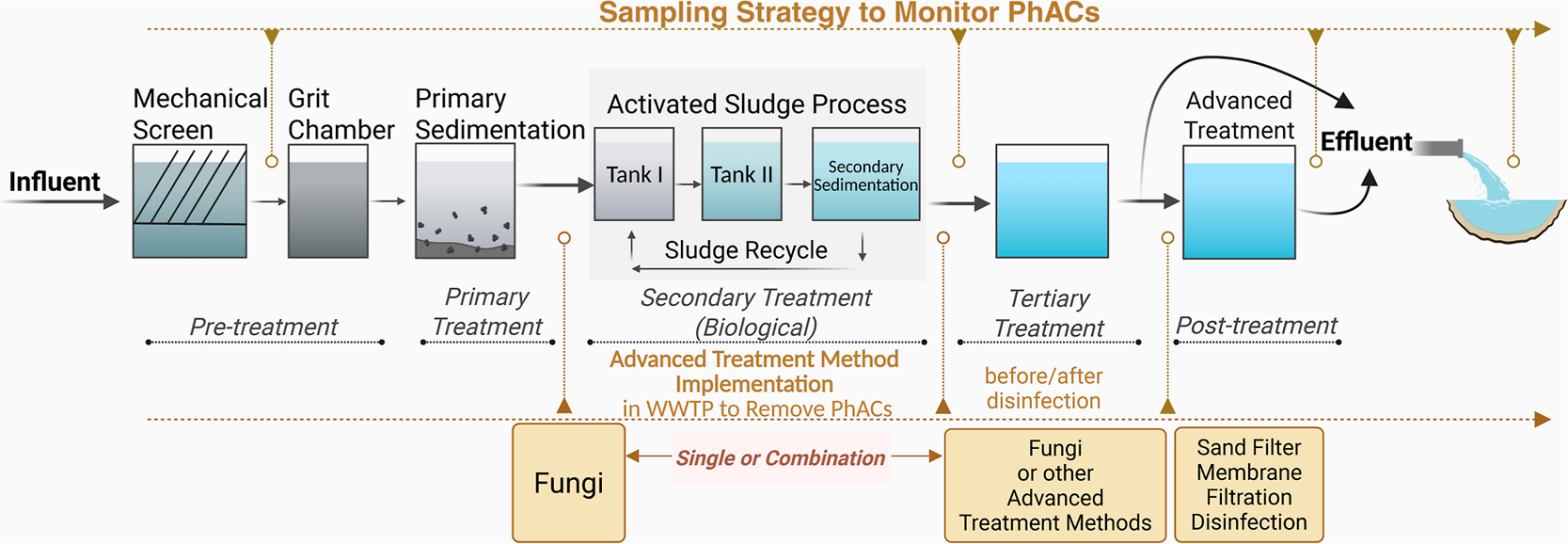
Conceptual framework for conventional WWTP processes: strategies for monitoring and advancing treatment methods implementation for PhACs.

To summarize, the advanced fungal technology for industrial-scale production that maintains sterility must be discarded, as it is neither economically nor environmentally feasible. Emphasizing nonsterile conditions enables a more realistic approach to fungi, accounting for adaptation and competition with native microorganisms. This shift in strategy can help bridge the gap between lab-scale success and real-world application, ultimately enhancing the practical implementation of fungal solutions in industrial wastewater treatment before it is discharged into the municipal sewer system.

## 8 Conclusions

Authors from this study have identified three main challenges in the role of filamentous fungi application in pharmaceutical bioremediation and wastewater management:

- Advancing treatment technologies: enhancing wastewater treatment plants by combining fungal treatments with advanced processes can improve pharmaceutical removal and address the limitations of existing systems.
- Challenges in sterile vs nonsterile conditions: most research on fungal performance is conducted in sterile conditions, with limited studies in real-world, nonsterile environments, emphasizing the need for practical application strategies.
- Research gap in LCA: no direct life-cycle assessment (LCA) studies exist for fungal systems, highlighting a critical gap in understanding their sustainability compared to conventional treatments.

The use of various advanced technologies in WWTPs can improve the treatment efficiency and environmental sustainability. While conventional methods reliably remove organic matter, advanced options such as AOP, AC, and membrane processes achieve higher removal rates for micropollutants, potentially reducing PhAC emissions from WWTPs by up to 69%. In this article, a shift in research strategies is proposed to address the practical requirements and challenges of real-world applications, ensuring that fungal solutions are both practical and effective in actual wastewater treatment scenarios. Finally, an LCA provides valuable insights into the sustainability, efficiency, and potential trade-offs of fungal-based treatments compared with conventional methods and is expected to encourage further research and stimulate greater interest in this field.

## Data Availability

During the preparation of this work, the authors used *OpenAI* to improve the readability and language of the manuscript. For research paper searching, the authors used *Consensus.App*. After using these AI tools, the authors reviewed and edited the content as needed and took full responsibility for the content of the published article.
